# Explosive radiation in high Andean *Hypericum*—rates of diversification among New World lineages

**DOI:** 10.3389/fgene.2013.00175

**Published:** 2013-09-11

**Authors:** Nicolai M. Nürk, Charlotte Scheriau, Santiago Madriñán

**Affiliations:** ^1^Department of Biodiversity and Plant Systematics, Centre for Organismal Studies Heidelberg, Heidelberg UniversityHeidelberg, Germany; ^2^Laboratorio de Botánica y Sistemática, Departamento de Ciencias Biológicas, Universidad de los AndesBogotá DC, Colombia

**Keywords:** adaptive radiation, Andes Mountains, rDNA ITS phylogenetics, Neotropics, Páramos, St. John's wort

## Abstract

The páramos, high-elevation Andean grasslands ranging from *ca*. 2800 m to the snow line, harbor one of the fastest evolving biomes worldwide since their appearance in the northern Andes 3–5 million years (Ma) ago. *Hypericum* (St. John's wort), with over 65% of its Neotropical species, has a center of diversity in these high Mountain ecosystems. Using nuclear rDNA internal transcribed spacer (ITS) sequences of a broad sample of New World *Hypericum* species we investigate phylogenetic patterns, estimate divergence times, and provide the first insights into diversification rates within the genus in the Neotropics. Two lineages appear to have independently dispersed into South America around 3.5 Ma ago, one of which has radiated in the páramos (*Brathys*). We find strong support for the polyphyly of section *Trigynobrathys*, several species of which group within *Brathys*, while others are found in temperate lowland South America (*Trigynobrathys* s.str.). All páramo species of *Hypericum* group in one clade. Within these páramo *Hypericum* species enormous phenotypic evolution has taken place (life forms from arborescent to prostrate shrubs) evidently in a short time frame. We hypothesize multiple mechanisms to be responsible for the low differentiation in the ITS region contrary to the high morphological diversity found in *Hypericum* in the páramos. Amongst these may be ongoing hybridization and incomplete lineage sorting, as well as the putative adaptive radiation, which can explain the contrast between phenotypic diversity and the close phylogenetic relationships.

## Introduction

High altitude mountain regions in the tropics constitute an open grassland vegetation type that is characterized by large rosette and small cushion plants, bunch grasses, and evergreen sclerophyllous shrubs (Luteyn, 1999). These high-elevation grasslands occur above the tree-line and below the upper limits of plant life “along the crests of the highest mountain ranges or on isolated mountaintops, like islands in a sea of forest” (Luteyn, 1999). The páramos of South America are discontinuously distributed along the Andean Cordilleras between *ca*. 2800–5000 m a.s.l. in Venezuela, Colombia, Ecuador, and northern Peru, with outliers in the Cordillera de Talamanca in Costa Rica and adjacent Panama.

A hyperdiverse high-elevation ecosystem has evolved since the northern Andes were formed in the Pliocene above the modern tree-line that marks the lower limit of páramo vegetation (*ca.* 3–5 Ma ago; Gregory-Wodzicki, 2000; Hoorn et al., 2010), which makes it one of the youngest of the Neotropical ecosystems (Graham, 2009). Today, the páramos alone comprise around 3500 vascular plant species (Luteyn, 1999; Sklenár et al., 2010; Graham, 2011a), half of which are of temperate origin at the generic level (Smith and Cleef, 1988; Sklenár et al., 2010; Weigend et al., 2010; Antonelli and Sanmartín, 2011a) as in other high elevation tropical floras (Gehrke and Linder, 2009). In contrast, the páramo flora is the richest overall tropical mountain flora and has the largest number of genera and endemic elements (Smith and Cleef, 1988; Myers et al., 2000).

High species richness in the tropical Andes has been attributed to their geographic extent and history (Graham, 2011a), the availability of migration routes for Holarctic lineages along mountain chains (Bell and Donoghue, 2005; Hughes and Eastwood, 2006; Antonelli et al., 2009), and drivers promoting rapid diversification, such as climatic oscillations during the Plio- to Pleistocene, habitat turnover and heterogeneity, and founder effects attributable to isolation on sky-island-like mountain formations (van der Hammen and Hooghiemstra, 2000; von Hagen and Kadereit, 2001; Luteyn, 2002; Hughes and Eastwood, 2006; Losos, 2010; Sklenár et al., 2010; Luebert et al., 2011). *Espeletia* (Cuatrecasas, 1986; Rauscher, 2002), *Gentianella* and *Halenia* (Kadereit and von Hagen, 2003), or *Lupinus* (Hughes and Eastwood, 2006; Drummond et al., 2012) are well known examples of adaptive radiations in the high-elevation Andean grasslands.

The flowering plant genus *Hypericum* (St. John's wort, Hypericaceae) is a prominent and often abundant component of the flora with arborescent shrubs in the sub-páramo (*H. laricifolium, H. irazuense*), dwarf shrubs in pastures or meadows from elfin forest to higher zones (*H. andinum, H. mexicanum, H. juniperinum*), or on rocky and disturbed places (*H. cardonae, H. humboldtianum*), and prostrate plants in damp areas in the grass páramo (*H. selaginella, H. prostratum*). Around 65 of the ~100 species described in *Hypericum* in South America are páramo endemics. Overall, *Hypericum* is of temperate origin and has its main center of species richness in the Old World (Nürk and Blattner, 2010; Meseguer et al., 2013; Nürk et al., 2013). Molecular phylogenetic studies using sequences of the nuclear rDNA internal transcribed spacers (ITS) (Meseguer et al., 2013; Nürk et al., 2013) and chloroplast data (Meseguer et al., 2013) revealed the three large *Hypericum* sections *Myriandra, Brathys* and *Trigynobrathys sensu* Robson (1977, 1987, 1990, 2012) as a monophyletic group, which includes ca. 90% of the *Hypericum* species native to the New World^1^, and verified the genus *Triadenum* to be included within *Hypericum* as sister to the *Myriandra*+*Brathys* s.l. clade of Nürk et al. (2013); i.e., clade B in Meseguer et al. [2013; see Ruhfel et al. (2011) for taxonomic implications].

*Hypericum* sect. *Myriandra* species are distributed mainly in the Nearctic with some located in Honduras, Bermuda and the Caribbean. The majority of New World *Hypericum* is classified in sects. *Brathys* and *Trigynobrathys* (Table 1). Although predominately a high-elevation group in the Neotropics, a small number of herbaceous *Hypericum* species has adapted to lower elevations, distributed below 3000 to less than 1000 m in lowland regions of temperate South America (Robson, 1977 onwards).

**Table 1:** **Sections of *Hypericum* (*sensu* Robson, 1977 onwards^a^) in this study, detailing species number and distribution**.

**Hypericum section**	**Species number**						
	**Total**	**North America**	**Central America**	**South America**	**Páramos**	**East Asia**	**Africa**
Triadenum^a^	6	4				2	
Myriandra	29	28	4^b^				
Brathys	90	2	15	73	63		
Trigynobrathys	59	13	9	27	4	5	5

a Triadenum is not included in the sectional classification of Hypericum (sensu Robson), but see Ruhfel et al. (2011).

b Only one species is endemic to Central America (H. limosum from Cuba).

**Figure 1:**
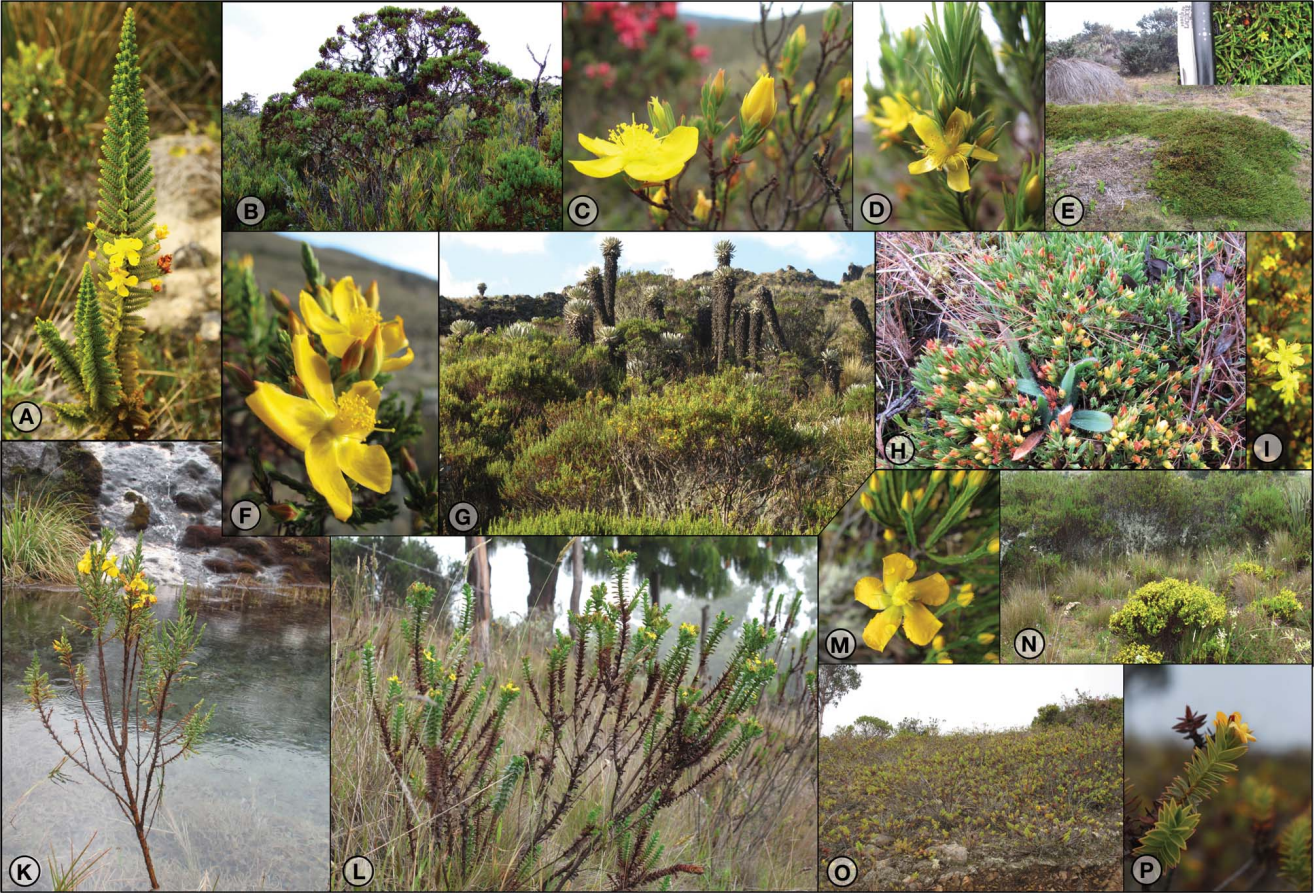
**Diversity in life forms and ecology in Andean high-elevation *Hypericum*. (A)** *H. woodianum*, 80–150 cm erect shrub, flowers 15–18 mm ∅, wet Andean forest to páramo; **(B)** *H. irazuense*, 70–500 cm shrub, flowers 25–30 mm ∅, open slopes or among bamboo in the páramos; **(C)** *H. garciae*, ca. 75 cm dwarf shrub, flowers 17–20 mm ∅, páramo, dry stony or sandy soil; **(D,I,N)** *H. juniperinum*, 20–100 (−250) cm erect (sub-) shrub, flowers 4–12 mm ∅, forest margins and damp or shaded subpáramo to páramo, often abundant in pastures/meadows; **(E)** *H. prostratum*, 10–30 cm prostrate shrub, flowers 4–8 mm ∅, open and wet páramo; **(F,G,K)** *H. laricifolium*, (10–) 180 (−600) cm shrub, flowers 15–30 mm ∅, open subpáramo to *Espeletia* páramo, well drained **(G)** to aquatic **(K)**; **(H)** *H. selaginella*, 5–15 cm prostrate shrub, flowers 6–8 mm ∅, dry and stony to dampish páramo; **(L)** *H. mexicanum*, 15–70 (−150) cm stiff erect shrublet, flowers 15–25 mm ∅, talus or grassy slopes to open páramo; **(M)** *H. tetrastichum*, 5–100 cm dwarf shrub, flowers 10–15 mm ∅, wet and dry often exposed areas in páramo; **(N)** *H. myricariifolium* (in the back, with *H. juniperinum* in the front), up to 200 cm tall shrub, flowers 18–25 mm ∅, open slopes in páramo; **(O,P)** *H. cardonae*, 20–100 cm shrub, flowers 12–18 mm ∅, dry rocky talus and cliffs. Panel **(1A)** by J. Paal, Panel **(1B)** by S. Crockett, Panel **(1P)** by G. Atchison.

While shrubs constitute the dominant life form in North America, a multitude of life forms evolved in the Neotropics—up to 6 m high sclerophylous arborescent to small dwarf shrubs, prostrate shrubs, and perennial to annual herbs. Most of this phenotypic diversity can be found in the páramos of the Andes (Figure 1). This diversity is mirrored by the great variety of ecological conditions occupied (for an overview see Crockett et al., 2010) and cytology; chromosome numbers range from (2n=) 8, 12, 16, 18, 22, 24, and 32 (Robson, 1987, 1990; Moraes et al., 2009), suggesting that polyploidization is involved in the evolution of *Hypericum* in South America. However, for only three species (*H. irazuense, H. silenoides, H. brasiliense*; multiple counts in the last) of Neotropical *Hypericum* have chromosome numbers been published (Robson, 1987, 1990; Moraes et al., 2009). This prevents comparative phylogenetic studies on the extent and distribution of polyploidy across the Neotropical species.

The recency of the páramo biome, the extent of ecological and phenotypic diversity (Figure 1), as well as high species richness (summarized in Robson, 2012) suggest that *Hypericum* could represent an adaptive radiation (Simpson, 1953) in the páramos of South America. Following Glor (2010), adaptive radiation is a response to natural selection and ecological opportunity involving diversification of species and associated adaptations. To define and diagnose adaptive radiation three operational criteria are considered in the phylogenetic context: (a) multiplication of species and common descent, (b) extraordinary diversification, and (c) adaptation via natural selection (phenotype-environment correlation and trait utility; Schluter, 2000; Sudhaus, 2004; Glor, 2010).

Insights into the evolutionary history of *Hypericum* in the Neotropics first demand phylogenetic hypotheses in an explicit framework to test questions about diversification rates and niche shifts, biogeography, key innovations, or polyploidization events (Harvey and Pagel, 1991; Emshwiller and Doyle, 1998; Pagel, 1999; Emshwiller and Doyle, 2002; Stephens and Wiens, 2003; Wiens and Donoghue, 2004; Moore and Donoghue, 2007, 2009; Crisp et al., 2011; Vamosi and Vamosi, 2011). The consideration of divergence times of lineages in the phylogenetic context is essential, as dispersal patterns and species richness are related to time (Ricklefs and Latham, 1992; Stephens and Wiens, 2003; Wiens and Donoghue, 2004), and area availability (Vamosi and Vamosi, 2010).

In a recent study, Meseguer et al. (2013) estimated divergence times and conducted biogeographic analyses based on chloroplast sequence variation (*trn*S*-trn*G, *trn*L*-trn*F, *psb*A-*trn*H) for the genus *Hypericum*. On the base of a rather reduced sampling of South American *Hypericum* (5 species, 7 accessions) they revealed a single Neotropical clade that had a mean crown group age of 3.9 Ma (Meseguer et al., 2013), sister to a clade containing North American and Asian species belonging to sect. *Trigynobrathys* (together called the “*Brathys*-group”). Furthermore, they suggest merging sect. *Trigynobrathys* into sect. *Brathys sensu* Robson (1977, 1987, 1990, 2012), as one species from sect. *Trigynobrathys* groups within a clade containing mainly species belonging to sect. *Brathys*, a result also revealed in phylogenetic studies analyzing morphological (Nürk and Blattner, 2010) and rDNA ITS data (Nürk et al., 2013).

In this study, we conduct new phylogenetic analyses and estimate divergence times in a Bayesian framework, using nuclear ITS sequences of a representative sampling of New World *Hypericum*. We employed a wide sampling across the New World species of *Hypericum* and a dense sampling within the Neotropics to expand prior phylogenetic hypotheses (Meseguer et al., 2013; Nürk et al., 2013) on *Hypericum* in South America. Despite potentially problematic issues resulting from the multi-copy nature of ITS (Baldwin, 1992; Baldwin et al., 1995) for phylogenetic inference (e.g., possible paralogs, and/or pseudogenes; Álvarez and Wendel, 2003; Bailey et al., 2003; Nieto Feliner and Rosselló, 2007) we used this marker system as it offers expanded species/accession sampling (Blattner, 1999; Nürk et al., 2013) while being aware of the potential challenges (see Discussion).

Based on a dense sampling within Neotropical *Hypericum*, we investigate phylogenetic relationships of the South American and especially the high-elevation Andean species. In particular, we aim to address the following questions: (1) Are Neotropical *Hypericum* species monophyletic, i.e., is there only one clade in the Neotropics, as suggested by Meseguer et al. (2013), or is there more than one? (2) Is the polyphyletic nature of sect. *Trigynobrathys* as reported in previous studies (Meseguer et al., 2013; Nürk et al., 2013) an artifact of species sampling? (3) Are ITS based divergence time estimations congruent with those previously reported from cpDNA (Meseguer et al., 2013)? (4) How many lineages colonized the páramos and what are the ages of these lineages? And finally, (5) are there differences in diversification rates between clades of New World *Hypericum*?

The first three questions examine previously published hypotheses and compare ITS age estimates to those inferred by analysis of chloroplast sequence variation. The latter two questions are a first attempt to diagnose adaptive radiation in Andean high-elevation *Hypericum*, by aiming at two of the three operational criteria that define adaptive radiation as proposed by Glor (2010), (a) multiplication of species and common descent, and (b) extraordinary diversification.

## Materials and methods

### Taxon sampling and molecular methods

Our approach involved broad sampling within New World *Hypericum* to ascertain hypotheses about backbone relationships (major lineages), as well as of closer related species from the Andean highlands. Samples were obtained from herbarium collections (ANDES, BM, GAT, HEID) and freshly collected silica-gel dried material. Forty-five ITS sequences selected from GenBank were included into the final data set, additionally to 135 sequences (representing 56 species) newly generated for this study (see Appendix, voucher), which have been submitted to the European Nucleotide Archive (http://www.ebi.ac.uk/ena/data/view/HG004646-HG004780, Accession No. HG004646–780). In total, the final data set contained 180 accessions (93 species), including 135 native to South America (56 species); a three- to ten-fold increase in species sampling in the Neotropics compared to previous studies (Meseguer et al., 2013; Nürk et al., 2013).

Genomic DNA was extracted with the Invisorb® Spin Plant Mini Kit (Stratec Molecular GmbH, Berlin, Germany) following the manufacturer's protocol. Amplification of the ITS region (including ITS-1, 5.8S rDNA, and ITS-2) followed the procedure detailed in Nürk et al. (2013), with separate amplification (and sequencing) of ITS-1 and ITS-2 for poorly preserved herbarium exsiccatae (Blattner, 1999). Cleaned amplification products were sent for sequencing to Eurofins MWG Operon (Ebersberg, Germany). Forward and reverse sequences from each template were manually edited and combined into a single consensus sequences with Geneious v5.4 (Biomatters, available from www.geneious.com). Sequences were checked for patterns in the chromatograms, which suggest multiple non-identical ITS copies (paralogs or pseudogenes), and multiple reads per site were coded as ambiguities (present in the spacer regions only, in 23 newly generated sequences with one to four ambiguous sites per sequence and in eight sequences downloaded from GenBank with one to seven ambiguous sites per sequence).

### Phylogenetic inference

Sequences were aligned using the L-INS-I algorithm implemented in the software Multiple Alignment using Fast Fourier Transform (MAFFT) v6.9 (Katoh et al., 2002; Katoh, 2005) and manually adjusted using PhyDE v0.9 (Available online: http://www.phyde.de). MrModeltest v2.3 (Nylander, 2004) was used to select the appropriate model of sequence evolution and the SYM + Γ model (Yang, 1993, 1994; Zharkikh, 1994) was chosen according to the Akaike Information Criterion (Akaike, 1974; Posada and Buckley, 2004). Phylogenetic analyses were performed under Maximum likelihood (ML; Felsenstein, 1981) and Bayesian Inference (BI; Mau et al., 1999). *Hypericum faurei* R.Keller [=*Triadenum japonicum* (Blume) Makino] belonging to the *Triadenum* clade was used as outgroup, following Nürk et al. (2013) who showed this clade to be sister to the rest. For ML analysis the RAxML GUI v1.1 (Stamatakis, 2006; Silvestro and Michalak, 2011) was used with the GTRCAT model and clade support was evaluated with 10000 rapid bootstrap pseudoreplicates (Stamatakis et al., 2008). For BI optimization MrBayes v3.2.1 (Ronquist and Huelsenbeck, 2003) was started with 4 independent runs, each with 4 chains for 10 million generations with the appropriate substitution model (SYM + Γ), setting temperature to 0.01, sampling every 1000 generations, and using the ML tree as a starting tree, but introducing random perturbations into the starting tree to initiate parameter calculation from different priors to enable detection of possible convergence problems (using the command “mcmcp nperts = 5”). Following the results of Meseguer et al. (2013), which showed a more realistic branch length estimation when introducing a lambda parameter correction in Bayesian phylogenetics on ITS sequence data in *Hypericum*, we used a ‘corrected’ exponential prior on branch length of 1/λ = 0.1 [“prset brlenspr = Unconstrained:Exp(100)”]. Convergence of the parameters was monitored using Tracer v1.5 (Rambaut and Drummond, 2007). After discarding 25% of the sampled trees as burnin, posterior probabilities were calculated on the BI stationary sample.

### Divergence time estimations

We choose the Bayesian tree to test for rate constancy among lineages. The likelihood scores associated with branch length were calculated on this tree in PAUP^*^ (Swofford, 2002) under the optimal model of sequence evolution and associated parameters with and without a strict molecular clock enforced. We followed the approach of Huelsenbeck and Rannala (1997) to assess significance. A global molecular clock was rejected (*p* < 0.05) for the rDNA sequence data. Therefore, divergence times were estimated under a relaxed molecular clock employing the uncorrelated lognormal (UCLN) model (Drummond et al., 2006) that assumes branch specific substitution rates to be drawn from a single lognormal distribution estimated from the data. Implementation of the UCLN model in BEAST v1.7.2 (Drummond and Rambaut, 2007) together with the use of Markov chain Monte Carlo (MCMC) sampling methods estimates both topology and substitution rates and calculates absolute divergence times and confidence intervals when calibrated with external data (fossils, or secondary calibration points like estimated ages revealed in other studies). The following calibration points were considered, relying on the age estimates reported in Meseguer et al. (2013). (1) The age of the root node estimated to (23.82−) 29.30 (−35, 23) Ma was constrained with a normal distribution that had a mean of 29, and a standard deviation of 5. (2) The age of the *Myriandra*+*Brathys* s.l. crown node estimated to (16.99−) 21.92 (−27, 33) Ma was constrained with a normal distribution that had a mean of 22, and a standard deviation of 4. (3) The age of the *Myriandra* crown node estimated to (9.41−) 13.59 (−18.48) Ma was constrained with a normal distribution that had a mean of 13.5, and a standard deviation of 3.

Analyses were performed in two independent runs in BEAST to test for convergence in divergence times, each consisted of 100 million generations, and sampling a tree every 10000 generation. Each run started from the tree obtained by ML search, after performing a semi-parametric method based on penalized likelihood (Sanderson, 2002) in R (R Development Core Team, 2013) with the “chronopl” command as implemented in the package APE (Paradis et al., 2004). The GTR model of nucleotide substitution was applied with the Γ model of site heterogeneity. The birth and death model of speciation considering incomplete sampling (Stadler, 2009) was set as tree prior. Accessions grouping in the BI tree within the *Myriandra* and *Myriandra*+*Brathys* s.l clades were constrained monophyletic. Convergence of the parameters was monitored using Tracer v1.5 (Rambaut and Drummond, 2007) and the resulting trees of the two runs were combined in LogCombiner (Drummond and Rambaut, 2007) with a burnin of 50%. Means and confidence intervals were calculated on the remaining 10002 trees in TreeAnnotator (Drummond and Rambaut, 2007) to obtain a final consensus tree [maximum clade credibility tree that has 95% of the highest posterior density (HPD)] for visualization in FigTree v1.3.1 (Rambaut, 2006–2013).

### Diversification rates

As approximation of net diversification rates (*r*) we used the simple macro-evolutionary constant rate, pure-birth (Yule model) taxonomic likelihood estimate of Magallón and Sanderson (2001) calculated as *r* = (ln *N*_1_ − ln *N*_0_)/*t*, where *N*_1_ = extant species (standing taxonomic diversity), *N*_0_ = initial species diversity, here taken as 1, and *t* = inferred clade age (time in Ma). We calculated *r* based on crown ages using the Bayesian mean and 95% HPD age estimates over the entire phylogeny, and clade-specific *r* for *Triadenum* (6 species), *Myriandra* (29 species), *Trigynobrathys* s.str. (≤52 species), *Brathys* (≥97 species), and the Páramo clade (≤67 species) and assigned species numbers according to species richness of the sections given in Robson (2012). The polyphyly of sect. *Trigynobrathys*, however, complicates the assignment of species richness to the *Trigynobrathys* s.str. and *Brathys* clades, as less than 30% of the species assigned to sect. *Trigynobrathys* are sampled in this study. Thus, we used the numbers given above, as we cannot approximate, which species belonging to sect. *Trigynobrathys sensu* Robson (1977 onwards) group within the *Brathys* and *Trigynobrathys* s.str. clade, respectively. For diversification rate estimation of the Páramo clade we assigned species richness in a conservative way, using only the number of species reported to be native to páramo habitats (Robson, 1987, 1990, 2012), i.e., used an underestimated number of species belonging to this clade.

To test the hypothesis of extraordinary diversification in the páramos, we applied the likelihood-based approach given in Magallón and Sanderson (2001). Specifically, we ask which of the major clades in our phylogeny are unexpectedly species-rich (or poor), given their age and the estimated net diversification rate for the entire phylogeny (i.e., the background rate for *Triadenum*+*Myriandra*+*Brathys* s.l.). We calculated 95% confidence intervals for the background net diversification rate (*r*) based on the crown group age using the formula *k*_upper_(*t*) = 1 + *log*β0.025 (1 + α) /*r* (1 − α − β + αβ) + α + 2β − 1 for the upper boundary value, and *k*_lower_(*t*) = 1 + *log*β0.975 (1 + α)/*r* (1 − α − β + αβ) + α + 2β − 1 for the lower, where β = (e^*rt*^ − 1/e^*rt*^ − ε) and α = εβ, and assuming (1) no extinction (relative extinction rate ε = 0), and (2) a reasonably high relative extinction rate (ε = 0.9). The 95% confidence interval of the expected number of species is the range of values between *k*_upper_ and *k*_lower_ in a semi-log plot for crown group ages of (log) species diversity vs. age at time *t* after the origin of a clade, under a given *r* and ε. Those clades that fall outside the confidence intervals are then regarded as being exceptionally species-rich or poor (Magallón and Sanderson, 2001).

## Results

### Phylogeny

**Figure 2:**
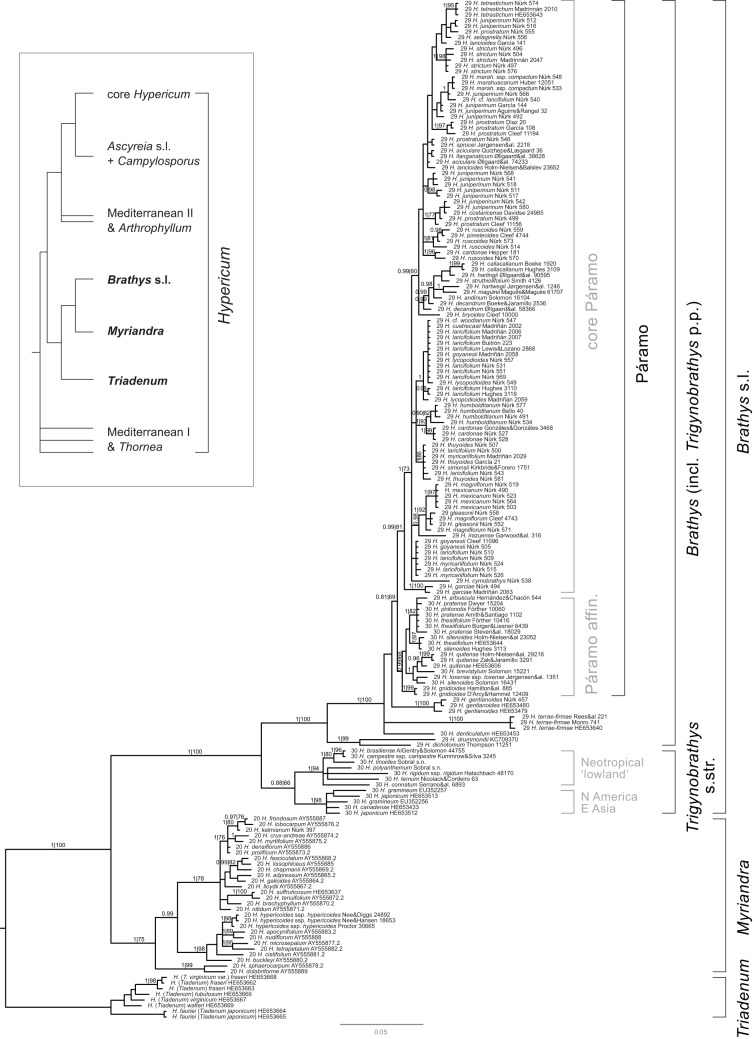
**Phylogeny of New World *Hypericum* inferred from rDNA ITS sequence Bayesian analysis.** In the tree sketch top left, the phylogenetic position of the species in this study within the genus *Hypericum* is marked by clade names in boldface. Bayesian posterior probabilities and ML bootstrap support is given above the branches (BPP|BS). Rooting is that of Nürk et al. (2013). Accession names consist of a section number (Robson, 2012), the species, and a collection/GenBank identifier. Clade names not considered in Figure 3B are given in gray.

The aligned data matrix comprised 755 characters, of which 366 were variable. The 50% majority-rule consensus tree of the Bayesian (BI) stationary sample (*n* = 30004 trees) (Figure 2) is highly congruent with the ML consensus tree (not shown) in the sense that there were no conflicts between strongly supported clades (>97% Bayesian posterior probability [BPP], >75% bootstrap support [BS]). Moreover, correction of the exponential prior (lambda) of branch length in the Bayesian analysis resulted in average branch length congruent with those inferred under ML. The trees and data sets produced in this study are available from TreeBase (http://www.treebase.org) study number 14179.

Relationships among the major clades (Figure 2) are consistent with previous studies (Meseguer et al., 2013; Nürk et al., 2013). North American sect. *Myriandra* form a clade (1.00 BPP, 75% BS), sister to *Brathys* s.l. (1.00 BPP, 100 BS), the latter containing all, but not solely Neotropical taxa. Species from East Asia and North America group within the *Trigynobrathys* s.str. clade, which is not strongly supported in our analysis (0.88 BPP, 66 BS), but present with good support in other studies (Meseguer et al., 2013; Nürk et al., 2013). Within *Trigynobrathys* s.str. two subclades are supported, one containing the East Asian-North American species (1.00 BPP, 98 BS), and the other containing Neotropical “lowland” species (i.e., native to lowland and upland areas of Brazil, Bolivia, Paraguay, Uruguay, and Argentina; 1.00 BPP, 94 BS). All other Neotropical species group within the *Brathys* clade (1.00 BPP, 100 BS), which also includes some species belonging taxonomically to sect. *Trigynobrathys*. This verifies sect. *Trigynobrathys sensu* Robson (1977 onwards) polyphyletic (non-monophyletic).

Within the *Brathys* clade, relationships are well-supported at the most basal dichotomy, but are almost without support between the remaining taxa. However, all species native to the páramos group within one clade (Figure 2), although without strong support (0.81 BPP, 69 BS). The species belonging to sect. *Trigynobrathys sensu* Robson (1977 onwards) group within one subclade of the Páramo clade (0.99 BPP, 68 BS) sister to the remainder. This subclade contains species from Central America (*H. pratense*) and from lowland areas of South America (*H. silenoides*), together with páramo natives (*H. gnidioides, H. arbuscula, H. thesiifolium*), the so-called Páramo affinis clade. The core Páramo clade (0.99 BPP, 81 BS) contains only species native to the high-elevation Andes. Apart from few subclades, which received moderate to strong support (e.g., a clade containing *H. cardonae* and *H. humboldtianum*, with 1.00 BPP, 93 BS), relationships are almost not resolved in the core Páramo clade.

### Age estimates and diversification rates

**Figure 3:**
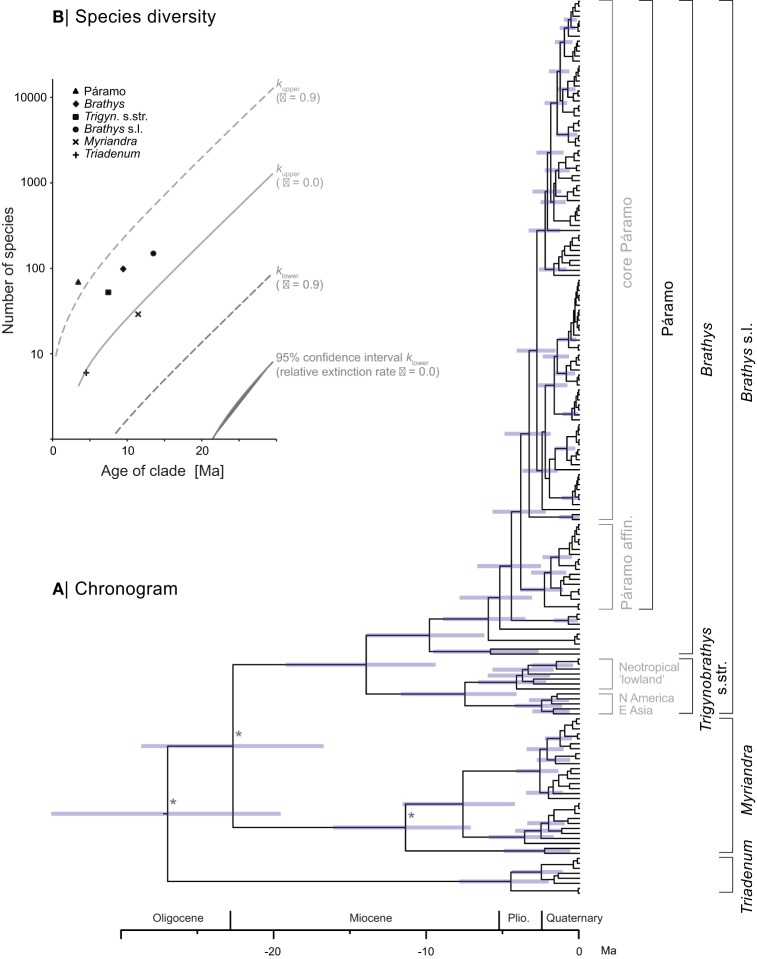
**Maximum clade credibility chronogram (A) of New World *Hypericum* produced from Bayesian divergence time estimations.** Asterisks mark nodes, which were time constrained for calibration. Clade names not considered in B are given in gray. **(B)** Test of exceptional species richness plotted for present species diversity (points) and expected (95% confidence interval, lines), assuming no extinction (solid lines, ε = 0) and high rates of extinction (dashed lines, ε = 0.9), based on crown ages. Note the species richness in the Páramo clade of *Hypericum* is higher than expected, under both high rates of extinction and no extinction.

The ultrametric time-calibrated maximum clade credibility tree (chronogram) obtained by the Bayesian relaxed clock analyses is shown in Figure 3A. The crown age estimates for the major clades are summarized in Table 2. The inferred divergence times of *Brathys* s.l. (this node was not constrained for calibration) is highly congruent with the ages reported by Meseguer et al. (2013). The same is true for the *Trigynobrathys* s.str. and the *Brathys* clade when compared to the ages revealed in Meseguer et al. (2013). According to our divergence time estimation, the subclade within *Trigynobrathys* s.str. containing the Neotropical “lowland” species diversified 4.1 Ma ago (2.2–6.5 95% HPD). The Páramo clade of *Hypericum* is 3.8 Ma old (2.3–5.6 95% HPD), with slightly younger estimates for the core Páramo clade (3.3 Ma, 1.9–4.8 95% HPD).

**Table 2:** **Crown group ages of major clades in New World *Hypericum* and net diversification rates, calculated on the given number of species for the mean Bayesian posterior age estimate, and the lower (*r*_Max_.) and upper (*r*_Min_.) 95% highest posterior density (HPD)**.

**Clade**	**Species number**	**Bayesian relaxed clock (crown ages)**			**Net diversification rate [*r*]**		
		**Mean**	**Lower 95% HPD**	**Upper 95% HPD**	**Mean**	**Max.**	**Min.**
*Triadenum+Myriandra+ Brathys* s.l.	184	26.77	19.57	34.41	0.195	0.266	0.152
*Triadenum*	6	4.48	2.07	7.78	0.400	0.866	0.230
*Myriandra*	29	11.35	7.15	16.02	0.297	0.471	0.210
*Brathys* s.l.	149	13.92	9.45	19.09	0.359	0.530	0.262
*Trigynobrathys* s.str.	52	7.47	4.17	11.59	0.529	0.948	0.341
*Brathys*	97	9.79	6.28	13.83	0.467	0.728	0.331
Páramo	67	3.83	2.26	5.62	1.098	1.860	0.748

Based on the ages obtained by Bayesian divergence time analysis, estimation of net diversification rates (*r*) under a Yule model and considering taxonomic richness reveals a higher rate for the Páramo clade of *Hypericum* [*r* = (0.75−) 1.10 (−1.86)] compared to all other clades (summarized in Table 2). The test of excessive species richness (Figure 3B) shows extraordinary species diversity in the Páramo clade when assuming a high relative extinction rate (ε = 0.9) for the entire phylogeny. The same is true when extinction is assumed to be zero (ε = 0.0). In the latter, however, all clades within the *Brathys* s.l. clade, that is, all clades containing Neotropical species, are revealed to be more species-rich than expected when compared to the background net diversification rate of the entire phylogeny.

## Discussion

Our analysis confirms the monophyly of *Myriandra* and *Brathys* s.l and corroborates the relationships among key lineages recovered in previous studies (Meseguer et al., 2013; Nürk et al., 2013). Four major clades, *Triadenum, Myriandra, Trigynobrathys* s.str., and *Brathys* comprise around 184 *Hypericum* species, which constitute ca. 90% of the species diversity of *Hypericum* in the New World (Robson, 2012). Nevertheless, it partially rejects the traditional infrageneric classification (Robson, 1977 onwards), revealing sect. *Trigynobrathys* (Robson, 1990) to be non-monophyletic^2^, a result reported also in other studies (Nürk and Blattner, 2010; Meseguer et al., 2013; Nürk et al., 2013). The extensive sampling in this study highlights the number of species, which group in a polyphyletic position.

What emerges from our analyses is striking evidence that Neotropical species, which group within two distantly related clades, underwent two independent biogeographic histories, as might be expected from their phylogenetic position. Moreover, both lineages exhibit different ecologies and life forms: one containing herbaceous species native to the low- and uplands of South America (*Trigynobrathys* s.str., subclade Neotropic “lowland”), the other mostly shrubby high Andean species (the Páramo clade). Thus, we assume that dispersal into South America occurred twice in two independent lineages within *Hypericum*. This is in contrast to Meseguer et al. (2013), who revealed a single dispersal to South America (which is due to restricted sampling of New World species in their analysis). Further conclusions demand formal biogeographic analyses on the base of a sound phylogeny with comprehensive species sampling, to reveal the spatiotemporal evolution of species and source areas of dispersals. Similarly, the inferred monophyly of the páramo species of *Hypericum*, and thus, the hypothesis of secondary evolution of “lowland” species (*H. silenoides*; placed in derived positions within the Páramo affinis clade) needs to be tested by ancestral area reconstructions incorporating altitudinal variation. Until then, monophyly of páramo *Hypericum* is the parsimonious hypothesis and the conditional diversification rates (see below) are conservative estimates.

Bayesian relaxed clock analyses of rDNA ITS revealed age estimates, which are in congruence to those inferred from analyses of cpDNA sequence divergence (Meseguer et al., 2013). To the best of our knowledge, this is the first report of divergence times in *Hypericum*, which have been estimated from nuclear data. According to these estimates (Figure 3A; Table 2), the stem lineages of the major clades, *Myriandra, Trigynobrathys* s.str., and *Brathys*, diverged during the Miocene (5.3–23.0 Ma ago), with an early split of *Triadenum* from *Myriandra*+*Brathys* s.l. in the Oligocene (23.0–33.9 Ma ago), and *Myriandra* from *Brathys* s.l. at the Oligocene/Miocene boundary (ca. 23 Ma ago). In contrast, the main diversification within the major clades is revealed to have taken place from the Pliocene (5.3 Ma) onwards (Figure 3). Within the Páramo clade of *Hypericum*, the inferred time of the onset of species divergence (ca. 3.3–3.8 Ma ago) coincides with the final uplift of the Andes, and thus, with the (early) emergence of the páramos (Gregory-Wodzicki, 2000; Hooghiemstra et al., 2006; Graham, 2009, 2011b). Moreover, the fossil record for *Hypericum* in Andean high valleys (pollen fossils; van der Hammen et al., 1973; Wijninga and Kuhry, 1990; Wijninga, 1996) reported for the Late Pliocene (2.5–3.6 Ma ago) is in good agreement with the revealed age estimates. Nevertheless, the used secondary calibration approach (calibrating the nodes of the tree with the estimated ages of another study) is problematic in the sense that the uncertainty produced in the original study is accumulated in our analysis.

Although topology and age estimates inferred from ITS sequence variation is highly congruent to those reported from chloroplast sequence analyses (Meseguer et al., 2013), the multi-copy nature of the 18S–ITS1–5.8S–ITS2–26S nuclear ribosomal cistron potentially confounds species tree reconstruction due to the possible presence of paralogs (derived from gene duplication) or pseudogenes (non-functional copies; Álvarez and Wendel, 2003; Bailey et al., 2003). Intra-individual rDNA polymorphism in *Hypericum* has been documented by Nürk et al. (2013), suggesting incomplete concerted evolution. If not artifactual, evidence for polymorphic ITS types has also been found in this study (indicated by double peaks in the chromatograms). On the other hand, the polymorphisms observed within an individual do not exceed that expected for a heterozygous individual (e.g., Muir et al., 2001). The direct sequencing approach used here, however, does not permit full investigation of ITS copy variation. While comparison with cpDNA phylogenetics (Meseguer et al., 2013) indicates no cases of ITS paralogy across major clades, the position of several species in multiple subclades of the Páramo clade (e.g., *H cardonae, H. juniperinum, H. prostratum*; Figure 2), could be evidence that divergent ITS lineages are present within individuals (deep paralogy *sensu* Bailey et al., 2003; duplication and divergence prior to speciation). Multiple ITS types within species could also result from hybridization and allopolyploidization (Emshwiller and Doyle, 1998, 2002; Blattner, 2004; Soltis et al., 2008; Kiefer and Koch, 2012). The sympatric occurrence of species (e.g., *H. mexicanum* with *H. juniperinum*, or *H. strictum* with *H. tetrastichum, H. prostratum*, and *H. selaginella*) provides the background in which hybridization is possible. Hence, gene trees and reticulate evolution could confound species relationships within the Páramo clade of *Hypericum*. Also, the possible presence of non-functional ITS copies (pseudogenes) could result in long branch attraction (Felsenstein, 1978), or repulsion (Siddall and Whiting, 1999). If present, pseudogenes affect divergence time estimations by accumulating substitutions that cause increased branch length estimates, which, in turn, result in earlier divergence time estimates. Comparison to future studies analyzing chloroplast sequence divergence or single/low copy genes and employing a comprehensive species sampling will reveal the influence of possibly included pseudogenes and the accuracy of the age estimates provided in the present study. Hence, ages reported here can be used as a conservative estimate, when implemented in diversification rate estimations.

We calculated net diversification rates (*r*), in which taxonomic richness of clades is considered (Table 2). The taxonomic likelihood approximation (Magallón and Sanderson, 2001) reveals a two-fold higher net diversification rate for the Páramo clade (mean: 1.1 speciation events per million year), compared to the *Trigynobrathys* s.str. clade (0.5 sp/Ma), i.e., the second group containing Neotropical species. The inferred rate of net diversification of páramo *Hypericum* is in the range reported from other high Andean plant groups, e.g., *Halenia* with 1.0 sp/Ma (Kadereit and von Hagen, 2003; von Hagen and Kadereit, 2003), *Gentianella* with 1.7 sp/Ma (von Hagen and Kadereit, 2001), or *Lupinus* with exceptional 1.9–3.7 sp/Ma (Hughes and Eastwood, 2006). When minimum and maximum rates of Páramo *Hypericum* are considered (0.75–1.86 sp/Ma), it is similar to the average net diversification rate of the páramos estimated as 1.36 sp/Ma (Madriñán et al., unpublished data).

That is, the Neotropical radiation in *Hypericum* seems to be related to the emergence of high-altitude habitats, as unexpected high species richness is mainly detected in the Páramo clade, and is less pronounced in the second Neotropical clade containing “lowland” species. We hypothesize that the availability of the “new” páramo habitats have had a causal impact on diversification of Neotropical *Hypericum*, allowing species to adapt into different niches within these newly emerging ecosystem and to rapidly diversify. A further possibility is that Pleistocene climatic fluctuations, repeated fragmentations of the “sky-island”-like páramo habitats promoted allopatric speciation leading to increased diversification (Rauscher, 2002; Hughes and Eastwood, 2006; Moore and Donoghue, 2007). Additionally, the diversification analysis is inference of acceleration from method of moments (Magallón and Sanderson, 2001), as the Yule model assumes constant rates over time. Punctual extinction or decreasing extinction rates might produce a similar pattern (Antonelli and Sanmartín, 2011b; Crisp and Cook, 2011; Stadler, 2013) that might not be associated to an increase in speciation in *Hypericum* in the páramos. These hypotheses need to be tested and compared to the influence of morphological, physiological, and spatiotemporal patterns to reveal causal cohesive motives underlying the observed extraordinary species richness (de Aguiar et al., 2009; Moore and Donoghue, 2009; Crisp et al., 2011; Stadler, 2011).

Further investigations on the impact of traits and/or events on diversification of páramo *Hypericum* demands a supported phylogeny for this group, which is not provided in the ITS data. Likewise, the amount and influence of hybridization and/or incomplete lineage sorting needs to be investigated by incorporation of both further nuclear and chloroplast markers (Rieseberg and Soltis, 1991; Jakob and Blattner, 2006; Carine et al., 2007). Comparison of nuclear low copy genes and cpDNA marker will offer to investigate the amount of reticulation, and incomplete lineage sorting/introgression (Peters et al., 2007; Nosil et al., 2009; Brassac et al., 2012) involved in diversification of *Hypericum* in the Andes.

To summarize, we conclude that only one lineage in *Hypericum* dispersed and diversified in the páramos. The age estimate for the Páramo clade of *Hypericum* correlates with the early emergence of high-elevation Andean grasslands. Based on these age estimates, extraordinary diversification is inferred for páramo *Hypericum*, as species numbers within these high-Andean grasslands are excessively rich. Great phenotypic diversity has evolved in a short time frame (3.3–3.8 Ma) in páramo habitats, although low genotypic differentiation is observed in the nuclear rDNA. Thus, keeping in mind the limitations discussed above, we propose that adaptive radiation—ecological and phenotypic diversity driven by intraspecific selection (on regulatory divergence rather than protein structure; Schluter, 2000; Losos, 2010; Mariac et al., 2010)—under strong ecological pressure caused the morphological diversity. That is, the rapid radiation in páramo *Hypericum* has likely been promoted by the uplift of the Andes, via adaptive radiation and/or (allopatric) speciation induced by the orogeny and topography of the northern Andean Cordilleras.

### Conflict of interest statement

The authors declare that the research was conducted in the absence of any commercial or financial relationships that could be construed as a potential conflict of interest.

## Footnotes

^1^The remaining 10% of the Hypericum species native to the New World, which are classified sensu Robson (1985, 2001, 2002, 2006) in sects. Umbraculoides (1 spec.), Roscyna (1 subsp.), Hypericum (1 native, 1 introduced spec.), Concinna (1 spec.), and Graveolentia (9 spec.), are distributed in North and Central America and belong phylogenetically to Hypericum clades remote from the large New World clade described above, and were thus, not included in this study.

^2^Hence, we refer with Brathys s.l. to sects. Brathys + Trigynobrathys sensu Robson (1977 onwards), and with Trigynobrathys s.str. to the East Asian-North American species (H. japonicum, H. gramineum, etc.) and the species native to lowland and upland areas of South America (H. brasiliense, H. connatum, etc.). Consequently, Brathys refers to sect. Brathys including sect. Trigynobrathys pro parte.

## Appendix

List of taxa included in the study of New World *Hypericum*. Information is given in the following order: Species, Voucher specimen/reference (for the sequences new to this study), ENA/GenBank ID.

***H. aciculare*** Kunth, B. Øllgaard, J.E. Madsen & L. Christensen 74233 (BM), HG004646; ***H. aciculare*** Kunth, W.W. Quizhepe & S. Lægaard 36 (BM), HG004649; ***H. adpressum*** W.P.C.Barton, AY555865.2; ***H. andinum*** Gleason, J.C. Solomon 16104 (BM), HG004725; ***H. apocynifolium*** Small, AY555883.2; ***H. arbuscula*** Stanley & Steyerm., H.M. Hernández & A. Chacón 544 (BM), HG004734; ***H. brachyphyllum*** (Spach) Steud., AY555870.2; ***H. brasiliense*** Choisy, Al Gentry & J.C. Solomon 44755 (BM), HG004770; ***H. brevistylum*** Choisy, J.C. Solomon 15221 (BM), HG004740; ***H. bryoides*** Gleason, A.M. Cleef 10000 (BM), HG004691; ***H. buckleyi*** M.A.Curtis, AY555880.2; ***H. callacallanum*** N.Robson, J.D. Boeke 1920 (BM), HG004726; ***H. callacallanum*** N.Robson, C.E. Hughes 3109 (HEID), HG004727; ***H. campestre*** subsp. ***campestre*** Cham. & Schltdl., R. Kummrow & J.M. Silva 3245 (BM), HG004771; ***H. canadense*** L., HE653433; ***H. cardonae*** Cuatrec., N.M. Nürk & G. Atchison 527 (ANDES), HG004689; ***H. cardonae*** Cuatrec., N.M. Nürk & G. Atchison 528 (ANDES), HG004690; ***H. cardonae*** Cuatrec., D.N. Hepper 181 (BM), HG004686; ***H. cardonae*** Cuatrec., F. & R. Gonzáles 3468 (BM), HG004688; ***H. chapmanii*** W.P.Adams, AY555869.2; ***H. cistifolium*** Lam., AY555881.2; ***H. connatum*** Lam., M. Serrano, J. Villalobos, A. lliully, J.A. Peñaranda & R. Lozano 6893 (BM), HG004774; ***H. costaricense*** N.Robson, G. Davidse 24985 (BM), HG004684; ***H. crux-andreae*** (L.) Crantz, AY555874.2; ***H. cuatrecasii*** Gleason, S. Madriñán 2002 (BM), HG004709; ***H. cymobrathys*** N.Robson, N.M. Nürk & G. Atchison 538 (ANDES), HG004743; ***H. decandrum*** Turcz., B. Øllgaard, S. Lægaard, K. Thomsen, J. Korning & T. Illum 58366 (BM), HG004730; ***H. decandrum*** Turcz., J.D. Boeke & J. Jaramillo 2536 (BM), HG004728; ***H. densiflorum*** Pursh, AY555886; ***H. denticulatum*** Walter, HE653453; ***H. dichotomum*** Lam., S.A. Thompson 11251 (BM), HG004760; ***H. dolabriforme*** Vent., AY555889; ***H. drummondii*** (Grev. & Hook.) Torr. & A.Gray, KC709370; ***H. fasciculatum*** Lam., AY555868.2; ***H. fauriei*** (***Triadenum japonicum***) R.Keller, HE653664; ***H. fauriei*** (***Triadenum japonicum***) R.Keller, HE653665; ***H.*** (***T. virginicum* var**.) ***fraseri*** (Spach) Steudel, HE653668; ***H.*** (***Triadenum***) ***fraseri*** (Spach) Steudel, HE653663; ***H.*** (***Triadenum***) ***fraseri*** (Spach) Steudel, HE653662; ***H. frondosum*** Michx., AY555887; ***H. galioides*** Lam., AY555864.2; ***H. garciae*** Pierce, N.M. Nürk & G. Atchison 494 (ANDES), HG004741; ***H. garciae*** Pierce, S. Madriñán 2063 (BM), HG004742; ***H. gentianoides*** (L.) Britton, Sterns & Poggenb., N.M. Nürk 457 (GAT), HG004757; ***H. gentianoides*** (L.) Britton, Sterns & Poggenb., HE653479; ***H. gentianoides*** (L.) Britton, Sterns & Poggenb., HE653480; ***H. gleasonii*** N.Robson, N.M. Nürk & G. Atchison 558 (ANDES), HG004750; ***H. gleasonii*** N.Robson, N.M. Nürk & G. Atchison 552 (ANDES), HG004752; ***H. gnidioides*** Seem., C. Hamilton, H. Stockwell & A. Aiello 885 (BM), HG004738; ***H. gnidioides*** Seem., W.G. D'Arcy & B. Hammel 12409 (BM), HG004739; ***H. goyanesii*** Cuatrec., A.M. Cleef 11096 (ANDES), HG004703; ***H. goyanesii*** Cuatrec., N.M. Nürk & G. Atchison 505 (ANDES), HG004704; ***H. goyanesii*** Cuatrec., S. Madriñán 2058 (BM), HG004714; ***H. gramineum*** G.Forst., EU352256; ***H. gramineum*** G.Forst., EU352257; ***H. harlingii*** N.Robson, B. Øllgaard, J.E. Madsen & S. Calderón 90595 (BM), HG004729; ***H. hartwegii*** Benth., P.M. JØrgensen, C. Ulloa, H. Vargas & P. Lozano 1246 (BM), HG004731; ***H. humboldtianum*** Steud., N.M. Nürk & G. Atchison 534 (ANDES), HG004674; ***H. humboldtianum*** Steud., N.M. Nürk & G. Atchison 577 (ANDES), HG004753; ***H. humboldtianum*** Steud., N.M. Nürk & G. Atchison 491 (ANDES), HG004755; ***H. humboldtianum*** Steud., M.A. Bello 40 (BM), HG004754; ***H. hypericoides*** subsp. ***hypericoides*** (L.) Crantz, G.R. Proctor 30665 (BM), HG004779; ***H. hypericoides*** subsp. ***hypericoides*** (L.) Crantz, M. Nee & B.F. Hansen 18653 (BM), HG004778; ***H. hypericoides*** subsp. ***hypericoides*** (L.) Crantz, M. Nee & G. Diggs 24892 (BM), HG004777; ***H. irazuense*** Kunze ex N.Robson, N. Garwood, M. Gibby, R.J. Hampshire & C.J. Humphries 316 (BM), HG004733; ***H. japonicum*** Thunb. ex Murray, HE653512; ***H. japonicum*** Thunb. ex Murray, HE653513; ***H. juniperinum*** Kunth, N.M. Nürk & G. Atchison 492 (ANDES), HG004654; ***H. juniperinum*** Kunth, N.M. Nürk & G. Atchison 566 (ANDES), HG004658; ***H. juniperinum*** Kunth, N.M. Nürk & G. Atchison 512 (ANDES), HG004672; ***H. juniperinum*** Kunth, N.M. Nürk & G. Atchison 516 (ANDES), HG004673; ***H. juniperinum*** Kunth, N.M. Nürk & G. Atchison 568 (ANDES), HG004675; ***H. juniperinum*** Kunth, N.M. Nürk & G. Atchison 541 (ANDES), HG004676; ***H. juniperinum*** Kunth, N.M. Nürk & G. Atchison 518 (ANDES), HG004677; ***H. juniperinum*** Kunth, N.M. Nürk & G. Atchison 511 (ANDES), HG004678; ***H. juniperinum*** Kunth, N.M. Nürk & G. Atchison 517 (ANDES), HG004679; ***H. juniperinum*** Kunth, N.M. Nürk & G. Atchison 542 (ANDES), HG004681; ***H. juniperinum*** Kunth, N.M. Nürk & G. Atchison 580 (ANDES), HG004682; ***H. juniperinum*** Kunth, C. García 144 (BM), HG004651; ***H. juniperinum*** Kunth, J. Aguirre & O. Rangel 32 (BM), HG004652; ***H. kalmianum*** L., N.M. Nürk 397 (GAT), HG004780; ***H.* cf. *lancifolium*** Gleason, N.M. Nürk & G. Atchison 540 (ANDES), HG004660; ***H. lancioides*** subsp. ***congestiflorum*** (Triana & Planch.) N.Robson, C. García 141 (BM), HG004653; ***H. lancioides*** subsp. ***lancioides*** Cuatrec., L. Holm-Nielsen & H. Balslev 23652 (BM), HG004680; ***H. laricifolium*** Juss., N.M. Nürk & G. Atchison 500 (ANDES), HG004697; ***H. laricifolium*** Juss., N.M. Nürk & G. Atchison 543 (ANDES), HG004701; ***H. laricifolium*** Juss., N.M. Nürk & G. Atchison 510 (ANDES), HG004705; ***H. laricifolium*** Juss., N.M. Nürk & G. Atchison 509 (ANDES), HG004706; ***H. laricifolium*** Juss., N.M. Nürk & G. Atchison 531 (ANDES), HG004716; ***H. laricifolium*** Juss., N.M. Nürk & G. Atchison 551 (ANDES), HG004717; ***H. laricifolium*** Juss., N.M. Nürk & G. Atchison 569 (ANDES), HG004718; ***H. laricifolium*** Juss., N.M. Nürk & G. Atchison 515 (ANDES), HG004722; ***H. laricifolium*** Juss., G.P. Lewis & P. Lozano 2868 (BM), HG004713; ***H. laricifolium*** Juss., S. Madriñán 2006 (BM), HG004710; ***H. laricifolium*** Juss., S. Madriñán 2007 (BM), HG004711; ***H. laricifolium*** Juss., X. Buitrón 223 (BM), HG004712; ***H. laricifolium*** Juss., C.E. Hughes 3110 (HEID), HG004720; ***H. laricifolium*** Juss., C.E Hughes 3119 (HEID), HG004724; ***H. linoides*** A.St.-Hil., M. Sobral s.n. (2007) (BM), HG004772; ***H. lissophloeus*** W.P.Adams, AY555885; ***H. llanganaticum*** N.Robson, B. Øllgaard, L. Holm-Nielsen, B. Boysen-Larsen, L.P. Kvist, A.R. Jensen & S. Wium-Andersen 38628 (BM), HG004650; ***H. lloydii*** (Svenson) W.P.Adams, AY555867.2; ***H. lobocarpum*** Gatt., AY555876.2; ***H. loxense*** subsp. ***loxense*** Benth., P.M. JØrgensen, C. Ulloa, H. Vargas & P. Lozano 1351 (BM), HG004735; ***H. lycopodioides*** Triana & Planch., N.M. Nürk & G. Atchison 557 (ANDES), HG004715; ***H. lycopodioides*** Triana & Planch., N.M. Nürk & G. Atchison 549 (ANDES), HG004719; ***H. lycopodioides*** Triana & Planch., S. Madriñán 2059 (BM), HG004721; ***H. magniflorum*** Cuatrec., N.M. Nürk & G. Atchison 519 (ANDES), HG004744; ***H. magniflorum*** Cuatrec., N.M. Nürk & G. Atchison 571 (ANDES), HG004749; ***H. magniflorum*** Cuatrec., A.M. Cleef 4743 (BM), HG004751; ***H. maguirei*** N.Robson, B. & C.K. Maguire 61707 (BM), HG004732; ***H. marahuacanum*** subsp. ***chimantaicum*** N.Robson, O. Huber 12051 (BM), HG004656; ***H. marahuacanum*** subsp. ***compactum*** (Triana & Planch.) N.Robson, N.M. Nürk & G. Atchison 548 (ANDES), HG004655; ***H. marahuacanum*** subsp. ***compactum*** (Triana & Planch.) N.Robson, N.M. Nürk & G. Atchison 533 (ANDES), HG004657; ***H. mexicanum*** L., N.M. Nürk & G. Atchison 490 (ANDES), HG004745; ***H. mexicanum*** L., N.M. Nürk & G. Atchison 523 (ANDES), HG004746; ***H. mexicanum*** L., N.M. Nürk & G. Atchison 564 (ANDES), HG004747; ***H. mexicanum*** L., N.M. Nürk & G. Atchison 503 (ANDES), HG004748; ***H. microsepalum*** (Torr. & A.Gray) A.Gray ex S.Watson, AY555877.2; ***H. myricariifolium*** Hieron., N.M. Nürk & G. Atchison 524 (ANDES), HG004707; ***H. myricariifolium*** Hieron., N.M. Nürk & G. Atchison 526 (ANDES), HG004723; ***H. myricariifolium*** Hieron., S. Madriñán 2029 (BM), HG004698; ***H. myrtifolium*** Lam., AY555875.2; ***H. nitidum*** Lam., AY555871.2; ***H. nudiflorum*** Michx., AY555888; ***H. philonotis*** Cham. & Schltdl., H. Förther 10060 (BM), HG004764; ***H. pimeleoides*** Planch. & Linden ex Triana & Planch., A.M. Cleef 4744 (BM), HG004693; ***H. polyanthemum*** Klotzsch ex Reichardt, M. Sobral s.n. (2007) (BM), HG004773; ***H. pratense*** Cham. & Schltdl., J. Amith & R. Santiago 1102 (BM), HG004765; ***H. pratense*** Cham. & Schltdl., J.D. Dwyer 15204 (BM), HG004763; ***H. pratense*** Cham. & Schltdl., W.D. Stevens, B.A. Krukoff & O.M. Montiel 18029 (BM), HG004768; ***H. prolificum*** L., AY555873.2; ***H. prostratum*** Cuatrec., A.M. Cleef 11194 (ANDES), HG004669; ***H. prostratum*** Cuatrec., A.M. Cleef 11156 (ANDES), HG004683; ***H. prostratum*** Cuatrec., N.M. Nürk & G. Atchison 546 (ANDES), HG004647; ***H. prostratum*** Cuatrec., N.M. Nürk & G. Atchison 555 (ANDES), HG004668; ***H. prostratum*** Cuatrec., N.M. Nürk & G. Atchison 499 (ANDES), HG004685; ***H. prostratum*** Cuatrec., A. Diaz 20 (BM), HG004670; ***H. prostratum*** Cuatrec., C. García 108 (BM), HG004671; ***H. quitense*** R.Keller, L. Holm-Nielsen, J. Jaramillo & F. Coello 29216 (BM), HG004736; ***H. quitense*** R.Keller, V. Zak & J. Jaramillo 3291 (BM), HG004737; ***H. quitense*** R.Keller, HE653606; ***H. rigidum*** subsp. ***rigidum*** A.St.-Hil., G. Hatschbach 48170 (BM), HG004775; ***H. ruscoides*** Cuatrec., N.M. Nürk & G. Atchison 570 (ANDES), HG004687; ***H. ruscoides*** Cuatrec., N.M. Nürk & G. Atchison 559 (ANDES), HG004692; ***H. ruscoides*** Cuatrec., N.M. Nürk & G. Atchison 573 (ANDES), HG004694; ***H. ruscoides*** Cuatrec., N.M. Nürk & G. Atchison 514 (ANDES), HG004695; ***H. selaginella*** N.Robson, N.M. Nürk & G. Atchison 556 (ANDES), HG004659; ***H. silenoides*** Juss., J.C. Solomon 16431 (BM), HG004762; ***H. silenoides*** Juss., L. Holm-Nielsen, J. Jaramillo & E. Bravo 23052 (BM), HG004761; ***H. silenoides*** Juss., C.E. Hughes 3113 (HEID), HG004769; ***H. simonsii*** N.Robson, J.H. Kirkbride & E. Forero 1751 (BM), HG004700; ***H. sphaerocarpum*** Michx., AY555878.2; ***H. sprucei*** N.Robson, P.M. JØrgensen, C. Ulloa & J. Caranqui 2218 (BM), HG004648; ***H. strictum*** Kunth, N.M. Nürk & G. Atchison 497 (ANDES), HG004661; ***H. strictum*** Kunth, N.M. Nürk & G. Atchison 576 (ANDES), HG004662; ***H. strictum*** Kunth, S. Madriñán 2047 (BM), HG004664; ***H. strictum*** Kunth, N.M. Nürk & G. Atchison 504 (ANDES), HG004663; ***H. strictum*** Kunth, N.M. Nürk & G. Atchison 496 (ANDES), HG004665; ***H. struthiolifolium*** Juss., D.N. Smith 4126 (BM), HG004756; ***H. suffruticosum*** W.P.Adams, HE653637; ***H. tenuifolium*** Pursh, AY555872.2; ***H. ternum*** A.St.-Hil., V. Nicolack & J.Cordeiro 63 (BM), HG004776; ***H. terrae-firmae*** Sprague & Riley, A.K. Monro 741 (BM), HG004759; ***H. terrae-firmae*** Sprague & Riley, R.G. Rees, K. Sidwell, G. Reid, R. Sundin & C. Bol 221 (BM), HG004758; ***H. terrae-firmae*** Sprague & Riley, HE653640; ***H. tetrapetalum*** Lam., AY555882.2; ***H. tetrastichum*** Cuatrec., N.M. Nürk & G. Atchison 574 (ANDES), HG004666; ***H. tetrastichum*** Cuatrec., S. Madriñán 2010 (BM), HG0S04667; ***H. tetrastichum*** Cuatrec., HE653643; ***H. thesiifolium*** Kunth, H. Förther 10416 (BM), HG004766; ***H. thesiifolium*** Kunth, W.C. Burger & R.L. Liesner 6439 (BM), HG004767; ***H. thesiifolium*** Kunth, HE653644; ***H. thuyoides*** Kunth, N.M. Nürk & G. Atchison 507 (ANDES), HG004696; ***H. thuyoides*** Kunth, N.M. Nürk & G. Atchison 581 (ANDES), HG004702; ***H. thuyoides*** Kunth, C. García 21 (BM), HG004699; ***H.*** (***Triadenum***) ***tubulosum*** Walter, HE653666; ***H.*** (***Triadenum***) ***virginicum*** L., HE653667; ***H.*** (***Triadenum***) ***walteri*** J.F.Gmel., HE653669; ***H.* cf. *woodianum*** I.M.Johnst., N.M. Nürk & G. Atchison 547 (ANDES), HG004708.
